# Efficacy and safety of selinexor in the treatment of AML: A protocol for systematic review and meta-analysis

**DOI:** 10.1097/MD.0000000000027884

**Published:** 2021-12-10

**Authors:** Liming Yu, Xuewei Yin, Yuping Si, Yan Wang, Jingyi Wang, Siyuan Cui

**Affiliations:** a The Third Affiliated Hospital of Shandong First Medical University, Jinan, Shandong Province, China; b First College of Clinical Medicine, Shandong University of Traditional Chinese Medicine, Jinan, Shandong Province, China; c Affiliated Hospital of Shandong University of Traditional Chinese Medicine, Jinan, Shandong Province, China.

**Keywords:** acute myeloid leukemia, meta-analysis, selinexor, systematic review

## Abstract

**Background:**

Acute myeloid leukemia (AML) is the most common leukemia among the adult population and accounts for about 80% of all cases. Despite advancements in therapeutic regimens, the prognosis remains very poor, especially in the elderly population. Selinexor is a first-in-class, oral, small molecule Exportin-1 inhibitor that is being developed for the treatment of a variety of cancers, including AML. The efficacy and safety issues of selinexor in the treatment of AML are still the focus of attention. Therefore, we conducted a meta-analysis to evaluate the efficacy and safety of selinexor in the treatment of AML.

**Methods:**

According to the search strategy, regardless of publication date or language, randomized controlled trials of selinexor for AML will be retrieved from 8 databases. First of all, the literature was screened according to the eligibility criteria, and use the Cochrane Collaboration's tool to assess the quality of the included literature. Then, using Rev Man 5.3 and STATA 14.2 software for traditional meta-analysis. Finally, the evaluation of the quality of the evidence and the strength of the recommendations will adopt the Grading of Recommendations, Assessment, Development, and Evaluation method.

**Results:**

This study will evaluate the efficacy and safety of selinexor for AML, thereby providing more evidence support for clinical decision-making in AML.

**Conclusion:**

Our research will provide more references for the clinical medication of patients with AML.

## 1 Introduction

Acute myeloid leukemia (AML) is a clinically heterogeneous disease characterized by accumulation and expansion of immature myeloid cells in the bone marrow and peripheral blood, with consequent failure of normal hematopoiesis.^[1]^ Despite improvements in AML therapy, relapse is still the most challenging aspect in AML. While 10% to 40% of younger AML patients are primarily refractory to AML induction therapy, the number is considerably higher for patients above 60 years (40%–60%).^[2]^ The cure rates have increased up to 15% in patients older than 60 years and about 40% in patients below 60 years of age only.^[3]^ So the prognosis remains very poor in the AML population. Still, the mainstay of treatment is chemotherapy, which has remained mostly unchanged over the past 4 decades. Therefore, extending the survival period of patients with AML, reducing the mortality rate, and improving the prognosis are key issues that need to be resolved.

Selinexor is a first-in-class, oral, small molecule inhibitor of Exportin-1 (XPO1), also known as chromosome maintenance protein 1, being developed for the treatment of cancer.^[4]^ XPO1 is the major nuclear exporter for tumor suppressor proteins (e.g., p53, p21, BRCA1/2, pRB, FOXO), growth regulators, and oncoprotein mRNAs (e.g., c-Myc, Bcl-xL, MDM2, cyclins), and is overexpressed in a variety of cancer cell types.^[5–7]^ Selinexor has orphan drug status in the USA and EU for AML, and is currently being evaluated in clinical trials in patients with AML.^[4]^ Therefore, this study proposes a systematic review program to evaluate the efficacy and safety of selinexor on AML, and to provide sufficient basis for further guidance of clinical medication, so as to avoid unnecessary traps.

## 2 Methods

### 2.1 Research registration

Our protocol has been registered on the International Platform of Registered Systematic Review and Meta-Analysis Protocols (INPLASY). The number was INPLASY2021100056 (URL = 10.37766/inplasy2021.10.0056). We will be based on the Preferred Reporting Items for Systematic Review and Meta Analysis Protocols (PRISMA-P), and strictly follow the requirements and conduct.^[8]^

### 2.2 Data sources and retrieval strategy

We will conduct a literature search from the following electronic databases: PubMed, EMBASE, the Cochrane Library, Web of Science, CNKI, Wan-fang Data, Chinese Biomedical Literature Database, Chinese Scientific Journal Database. There are no restrictions on publication date and language. In addition, the references listed in each included article are also manually searched.

Retrieve the databases by combining subject words with random words. Appropriate adjustments will be made according to the grammatical rules of different databases to ensure the completeness and comprehensiveness of the search. We will firstly conduct a pre-search, and discuss the problems encountered in the search process with the team. After confirming that there are no problems, we will conduct a formal literature search. Taking PubMed as an example, the retrieval strategy is shown in Table 1.

**Table 1:** Retrieval Strategy for PubMed.

Number	Search item
#1	Acute myeloid leukemia [Mesh]
#2	Acute myeloid leukemia [Title/Abstract] OR AML[Title/Abstract]
#3	#1 OR #2
#4	Selinexor [Mesh]
#5	Selinexor [Title/Abstract] OR Exportin-1 inhibitor [Title/Abstract] OR XPO1 inhibitor [Title/Abstract]
#6	#4 OR #5
#7	randomized controlled trial[Title/Abstract] OR controlled clinical trial[Title/Abstract] OR RCT[Title/Abstract] OR randomized[Title/Abstract] OR randomly[Title/Abstract]
#8	#3 AND #6 AND #7

### 2.3 Eligibility criteria

We will formulate the inclusion and exclusion criteria for this study based on the PICOS principles.

#### 2.3.1 Participants

Patients with AML diagnosed by WHO criteria diagnosis of AML will be included. There are no restrictions on nationality, age, sex, or race. Patients with severe liver and kidney, or other uncontrolled systemic diseases are excluded.

#### 2.3.2 Interventions and comparators

The recommended phase II dose (RP2D) of selinexor was 60 mg twice weekly in the treatment group. The control group was only given routine chemotherapy, or the same dose of placebo was given on the basis of routine chemotherapy. Routine chemotherapy regimens mainly include cytarabine plus idarubicin, high-dose cytarabine and mitoxantrone, decitabine, cytarabine, etoposide and mitoxantrone, cytarabine, cladribine and granulocyte-colony stimulating factor, cytarabine and fudarabine, etc.^[9–12]^

#### 2.3.3 Outcomes

The primary outcomes include ORR, CR, Cri, and DOR; the secondary outcomes include decrease in bone marrow blasts from baseline, quality of life, etc; the safety indicators include thrombocytopenia, fatigue, nausea, anemia, decreased appetite, decreased weight, diarrhea, vomiting, hyponatremia, neutropenia, leukopenia, constipation, and other adverse events.

#### 2.3.4 Type of studies

Randomized controlled trials (RCTs) will be included in this study irrespective of language or publication category. Animal trials, review article, and studies with incorrect RCT designs will be excluded.

### 2.4 Literature screening and data extraction

**Figure 1:**
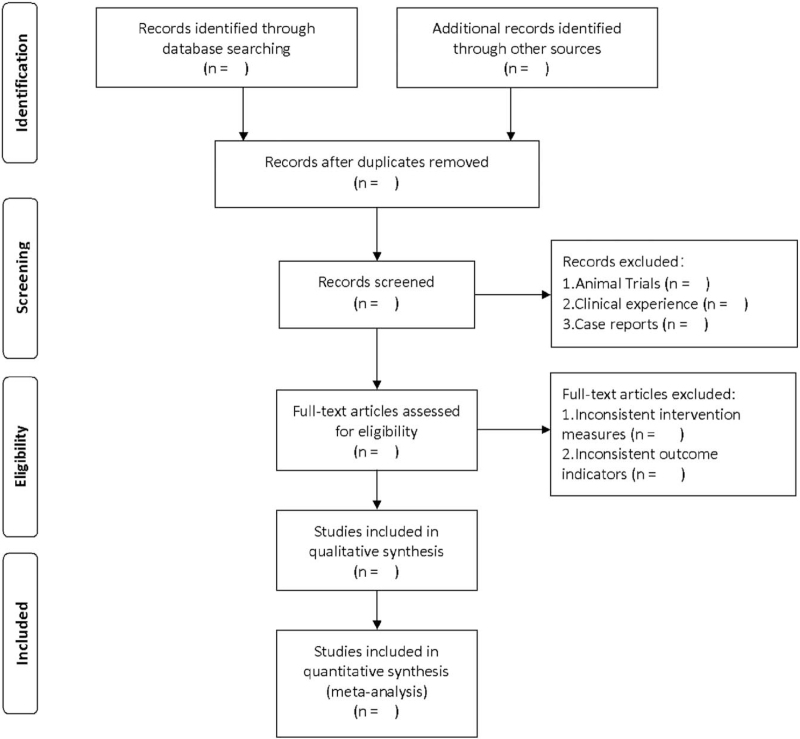
Flow diagram of literature retrieval.

Use Endnote X9.0 software to manage literature. After searching literature based on the above steps, import them into endnote software for literature screening. First, 2 independent researchers will conduct a preliminary literature screening based on the titles and abstracts of the included literature to eliminate duplicate and non-RCTs. Then read the full text of the remaining literature according to the previously designed principles of eligibility criteria, and finally determine the appropriate literature. When 2 researchers disagree, a third researcher will resolve it. The specific screening process is shown in Figure 1.

According to the Cochrane Handbook for Systematic Reviews of Interventions, 2 researchers independently extracted and recorded the required information from all the included literature. When 2 researchers disagree, they will discuss to reach an agreement, otherwise they will work with the third researcher to resolve. The required information mainly includes the author, publication time, study design, participants number and demographic characteristics (age, sex, etc), treatment status (e.g., the initial dose and treatment period of selinexor), and outcomes. If any of the above information in the included literature is incomplete, we will contact the corresponding author via email to obtain the required data.

### 2.5 Quality assessment

Two reviewers will independently assess the quality of the included literature according to the Cochrane Collaboration's tool for RCTs. If there is a disagreement between 2 reviewers, the third reviewer resolves the issue. According to Cochrane Handbook V.5.2.0, characteristics of each item will be evaluated in 3 categories: low, unclear, and high.^[13]^ The results of the quality assessment will be completed using software Review Manager 5.3.

### 2.6 Statistical analysis

#### 2.6.1 Traditional meta-analysis

We will execute Rev Man 5.3 and STATA 14.2 software for traditional meta-analysis. For dichotomous data, we will calculate a summary estimate with 95% confidence interval odds ratio value; for continuous data, we will calculate a summary estimate of standardized mean difference with 95% confidence interval, and *P* < .05 is considered statistically significant. The heterogeneity among the included literature will be assessed using the Q test method and I^2^ statistic method. When the Q statistic corresponds to *P* ≤ .10 or I^2^ > 50%, it indicates that there is heterogeneity among the included literature, and assess the effect size by the random effect; on the contrary, a fixed effect model is used.

#### 2.6.2 Subgroup analysis

Taking into account the issue of heterogeneity, we will conduct a subgroup analysis based on the specific circumstances of the included literature. If there is a problem of heterogeneity, we will conduct a subgroup analysis of age, sex, and interventions.

#### 2.6.3 Sensitivity analysis

This systematic review will use the method of eliminating each study one by one for sensitivity analysis. If the effective indicators of selinexor in the treatment of AML have not changed significantly, it indicates that the study is robustness. On the contrary, it is not robustness. According to the specific situation, low-quality research is excluded.

#### 2.6.4 Publication biases

Publication biases will be assessed by a funnel plot for meta-analysis and quantified by the Egger method. It should be noted that if the number of included literature is ≥10, it is appropriate to use a funnel plot to assess potential publication bias. However, if the included literature is <10, it may affect the overall test power because the included number is too small, and it is difficult to accurately evaluate the symmetry of the funnel plot.

### 2.7 Evidence quality assessment

The Grading of Recommendations, Assessment, Development, and Evaluation used to assess the quality of evidence. The quality of evidence is divided into 4 levels from 5 aspects, namely high, medium, low, and very low.^[14]^

### 2.8 Ethical considerations and dissemination plans

This study is a systematic review, it does not involve medical ethics and patients’ informed consent. This study will publish the results of meta-analysis in journal papers.

## 3 Discussion

AML is a form of cancer that is characterized by infiltration of the bone marrow, blood, and other tissues by proliferative, clonal, abnormally differentiated. In spite of advances, including allogeneic stem cell transplantation and a growing molecular tailoring of treatment toward driver genes such as FLT3,^[15]^ the prognosis of relapsed or refractory AML remains poor. With high relapse rates and few targeted therapeutic options, there is a need to develop novel solutions for the treatment of AML.

Selinexor selectively inhibits XPO1 by forming a slowly reversible covalent bond with cysteine 528 in the cargo binding pocket of XPO1.^[7]^ Selective inhibition of nuclear export by selinexor results in accumulation of tumor suppressor proteins in the nucleus, decreased levels of oncoproteins, cell cycle arrest, and apoptosis of cancer cells, while sparing normal cells.^[5–7]^ Selinexor has been shown to be effective in many clinical trials of AML. Selinexor plus cytarabine plus idarubicin produced an ORR of 55% (n = 42) in patients with relapsed or refractory AML in a phase II trial (NCT0224909).^[9]^ Perhaps it can bring hope to the treatment of AML.

Although this systematic review has many advantages, such as statistical power and accuracy of effect size estimation, subgroup analysis of factors affecting treatment effects, a more objective evaluation of evidence and a more accurate and objective evaluation of effect indicators evaluation. However, it also has some problems. For example, this study is an evaluation of published literature, and there may be problems such as unscientific and non-strict RCT design, resulting in uneven quality of literature research, which affects the credibility of this study. Or the research results included in the literature have false negatives and false positives. In any case, this systematic review can provide reliable results for AML, and provide strong evidence for the significant advantages of selinexor in the treatment of AML.

## Author contributions

**Conceptualization:** Liming Yu, Siyuan Cui.

**Data curation:** Xuewei Yin.

**Formal analysis:** Yuping Si.

**Funding acquisition:** Siyuan Cui, Liming Yu.

**Methodology:** Yan Wang, Jingyi Wang.

**Project administration:** Siyuan Cui.

**Writing – original draft:** Liming Yu, Siyuan Cui.

**Writing – review & editing:** Liming Yu, Xuewei Yin.
